# Aligning Federated Learning with Existing Trust Structures in Health Care Systems

**DOI:** 10.3390/ijerph20075378

**Published:** 2023-04-03

**Authors:** Imrana Yari Abdullahi, René Raab, Arne Küderle, Björn Eskofier

**Affiliations:** Machine Learning and Data Analytics (MaD) Lab, Department Artificial Intelligence in Biomedical Engineering (AIBE), Friedrich-Alexander-Universität (FAU) Erlangen-Nürnberg, 91054 Erlangen, Germany

**Keywords:** federated machine learning, health care system, patient-centered health, mobile health, COVID-19 proximity tracker, edge computing, peer-to-peer, security, privacy

## Abstract

Patient-centered health care information systems (PHSs) on peer-to-peer (P2P) networks (e.g., decentralized personal health records) enable storing data locally at the edge to enhance data sovereignty and resilience to single points of failure. Nonetheless, these systems raise concerns on trust and adoption in medical workflow due to non-alignment to current health care processes and stakeholders’ needs. The distributed nature of the data makes it more challenging to train and deploy machine learning models (using traditional methods) at the edge, for instance, for disease prediction. Federated learning (FL) has been proposed as a possible solution to these limitations. However, the P2P PHS architecture challenges current FL solutions because they use centralized engines (or random entities that could pose privacy concerns) for model update aggregation. Consequently, we propose a novel conceptual FL framework, CareNetFL, that is suitable for P2P PHS multi-tier and hybrid architecture and leverages existing trust structures in health care systems to ensure scalability, trust, and security. Entrusted parties (practitioners’ nodes) are used in CareNetFL to aggregate local model updates in the network hierarchy for their patients instead of random entities that could actively become malicious. Involving practitioners in their patients’ FL model training increases trust and eases access to medical data. The proposed concepts mitigate communication latency and improve FL performance through patient–practitioner clustering, reducing skewed and imbalanced data distributions and system heterogeneity challenges of FL at the edge. The framework also ensures end-to-end security and accountability through leveraging identity-based systems and privacy-preserving techniques that only guarantee security during training.

## 1. Introduction

### 1.1. Motivation

Electronic health records (EHRs) are commonly institution-specific. Consequently, the stored health information is isolated, fragmented, and duplicated across providers and patients may lack complete access to their medical histories. As a solution, countries such as Lithuania and Norway are adopting nationwide EHRs for their health care systems [1]. This way, the health information is well managed and digitally connected to avoid duplicated records and improve the quality and co-effectiveness of medical care as well as patient safety [2]. As specified by the German Health Care Information Technology Infrastructure (HTI), their new EHR should store complete medical histories of patients such as previous diagnoses, therapeutic decisions, treatment reports, and self-measurement values [2,3,4,5]. Among other benefits, patients should have autonomy to select freely between EHR providers (e.g., hospitals and insurance companies), hold data sovereignty for their EHR, and withdraw access rights at any time. 

In line with these principles, patient-centered health care information systems (PHSs) emerged and are defined as scalable systems that complement the traditional EHR, fulfill the HTI specifications, and offer new functionalities (e.g., translation of clinical information into layman’s terms or provision of vetted information to support self-administered interventions) [1,6,7]. They store health records (locally or remotely) under the control of data owners (patients) to empower and increase patients’ value, reduce health care transaction costs, and ensure collaboration between various health care stakeholders [1,8]. PHSs could be deployed on centralized or distributed ledger technologies (DLT); however, PHSs on less rigid and flexible peer-to-peer (P2P) networks store data locally under the sovereignty of individual device owners. They are inexpensive, resilient, transparent, and fit well with the large-scale efforts to re-decentralize the internet (e.g., Tim Berners-Lee’s Solid project [9]). Synergies between flexible and less rigid P2P and DLT-based technologies are known [1,10]. However, such technologies are distinct; for instance, on DLT, references or information are immutable and inherently distributed to all parties on the network (this is problematic for health care data due to privacy and data regulations), while on flexible P2P, information is stored locally and is logically and physically decentralized [1]. Nevertheless, this study focuses on less rigid P2P PHSs such as the e-toile framework in Switzerland [11], Doc.AI [12], and OnePatient [13], or disease-focused PHSs such as decentralized systems for Bluetooth-based monitoring of SARS-CoV-2 (or COVID-19) [14,15]. Such increased privacy management property of P2P networks spurs their strength and growing usage; e.g., Corona Warn App had 6.4 million downloads the day after its launch in 2020, growing to 45.05 million in the first quarter of 2022 [16]. These developments simplify the technical and organizational challenges to implementing data regulations such as the General Data Protection Regulation (GDPR) of the European Union [17].

The P2P PHS paradigm raises concerns regarding security and usability, for example, because of the absence of a “powerful” centralized or distributed node to serve as a trusted computing base on the network [1,18]. They also weaken the expectation for “active” involvement of other significant health care stakeholders since only patients have copies of their data. In the current health care system, patients interact with their practitioners, insurance companies, and the government (as primary identity providers), among others. Legally, health care practitioners are even required to store a copy of their patients’ data for up to 8 years [19]. This trust structure is unlikely to change in the coming decades; therefore, P2P PHSs’ concept of sidelining the current health care workflow could impede their adoption—e.g., how incapacitated patients’ health data could be accessed in case of an emergency. Moreover, the interaction between AI algorithms and practitioners needs to be considered in modern health care systems. Effective performance of such algorithms is crucial for using health care applications; e.g., practitioners were found to not trust an AI-based health tool (IBM Watson for Oncology) deployed to assist them in diagnoses for common reasons: during the simulation, the app mistakenly prescribed a medication that occasionally could kill a patient [20]. This is likely due to practitioners having no control of the diagnostic criteria of the model or the absence of aligning practitioners’ needs in the application life cycle.

P2P PHS provides an interoperable health care ecosystem to share and maintain data from multiple providers and sources, such as wearable sensors and health records at the hospitals. Such an ecosystem allows using more commonly found data from distinct domains in real-world applications to train machine learning (ML) models. In contrast, the P2P PHS approach puts data in isolated, heterogeneous, and distributed environments; it poses new challenges to the conventional data transaction procedures employed in ML today [21]. The traditional procedures for acquiring Big Data in ML involve several parties collecting the data, transferring them to a central data repository, and fusing them to build a model, whereas the data owners may be unclear about these procedures and the model’s future use cases. For that reason, it may violate laws such as the GDPR [17]. To address these challenges, federated learning (FL) approaches can be leveraged to build ML models that do not need data to be stored centrally but instead can be trained on the local devices that store the data. In this manner, only the model updates are returned to the central server.

### 1.2. Problems and Objectives

To reach a target learning accuracy, each phase of FL (Section 2.2) requires adequate iterative and frequent communication to exchange information between the server and the participants. Such required communication efficiency by the FL is affected on P2P PHSs: (i) P2P PHSs are provisioned on available patients’ devices, which are heterogeneous and are constrained by limited bandwidth resources and space. This introduces communication issues. For instance, clients’ model updates are delayed due to lack of or low bandwidth—if the aggregation engine waits for updates from stragglers, some edge clients become idle. Therefore, the FL training process, speed, and model personalization could be affected. (ii) P2P PHSs have heterogeneous data structures—different practitioners treating patients with different diseases and symptoms. When training on such highly imbalanced data and skewed distribution (e.g., patients’ health data varies according to disease type, age, or location), FL algorithms typically need more training rounds (more communications to the edge) to achieve a model with higher accuracy and convergence rates. As such, model accuracy is decreased when a local model only sees a subset of classes during training.

These challenges can be addressed currently by (i) minimizing the cost concerning non-uniform data distribution and FL statistical challenges at the edge using solutions such as consensus (e.g., sampling of local model updates [20]) or pluralistic (e.g., capturing the task relatedness using graph methods through multi-task learning [20]); (ii) reducing the number of model updates, dropping clients that did not meet the local update submission deadline, or using compression techniques (e.g., sparsification [20]) could improve communication efficiency in FL, although such solutions could yield performance penalty and information loss. Despite that, the use of FL in P2P PHSs introduces additional challenges due to their novelty and architectural designs. For example, as opposed to the typical FL process, on a typical highly decentralized P2P PHS network architecture, there is no “powerful” single centralized node to orchestrate the training process and perform model update aggregation. Therefore, the use of traditional FL could run into scalability issues if adopted on P2P PHSs that have multi-tier structures and hybrid design patterns [1,13].

There are efforts in the literature [15,22,23,24,25,26] that establish hierarchical design settings as proposed for P2P PHS [1] (e.g., clustering information based on their confidentially or random selection of devices and then assigning them to some hierarchy) to make FL scalable with enhanced communication efficiency. However, such solutions are based on “fully” centralized architectures in which trust is implicit and are not suitable for systems that have underlying design patterns like the PHS [1,13] (the architecture is detailed in Section 2.3). For example, if the existing schemes [15,23,24,25,26] are directly used for P2P PHS [1], other nodes in the architecture tier, such as practitioners’ nodes, could be rendered useless, although their abilities could be leveraged, e.g., to ease access to patients’ data. Furthermore, existing health care systems have different ways of managing health records, and workflows vary from stakeholder to stakeholder, including practitioners, hospitals, etc. To ensure the usability and utility of health care applications such as the emerging P2P PHSs, it is crucial to align and integrate every significant health care stakeholder and their objectives in the system. This study direction also focuses on keeping P2P PHS networks’ core values and reducing their barriers to the use of FL by taking a step away from “fully” decentralized networks to increase their adoption across the health domain. 

On account of existing studies, [15,20,22,23,24,27], this study proposes a novel FL conceptual framework, CareNetFL, based on existing health care system trust structures that are suitable for the emerging P2P PHS multi-tier structure and hybrid architecture (Section 2.3) with increased trust, scalability, and communication efficiency. Further, we discuss the proposed FL framework as a solution to address FL challenges related to P2P PHS (Section 4), such as concern with adoption and usability due to non-alignment with the current health system, being unfit for multi-tier and hybrid models, having limited network resources/space, and non-IID and system issues due the heterogeneity of P2P PHSs. CareNetFL has model life cycle stages (Section 3) that are scalable for FL on hierarchy and hybrid system architectures and flexible to be adopted by providers who want to implement FL for P2P PHSs while maintaining trust, security, and FL performance. Our study has the following implications for research and practice. 

Unlike state-of-the-art FL frameworks [15,20,22,23,24,27] that use random entities (that could be malicious) for model updates’ aggregation (Section 2.1), CareNetFL leverages entrusted roles such as practitioner nodes in the P2P PHS architectural hierarchy as a medium for requesting model training on the patients’ local storage. Practitioner nodes also function as heterogeneous data handlers to improve FL communication efficiency and privacy. This design conserves fundamental PHS features while allowing model training since learning orchestrators (e.g., a researcher) have neither access to nor direct communication with the patients on the PHS network. Patients are grouped and associated with their practitioners (e.g., dementia patients to neurologists) in CareNetFL. This brings data close to a uniform distribution, mitigates imbalanced data distribution issues, and reduces the required communication rounds to train an FL model with higher accuracy at the edge. Therefore, CareNetFL supplants the existing techniques such as compression [20]—which suffers from information loss, personalization issues, etc. [20]—when used to address data heterogeneity issues for FL at the edge. Including medical practitioners in the FL process allows them to oversee the model’s diagnostic criteria and address the barriers (missing measurements, etc.) to using patient-generated data across clinical settings. Moreover, our FL concepts provide a foundation that offers insights into how identity-based systems could be leveraged to provide end-to-end confidentiality, integrity, and accountability to the data and model for the entire training life cycle—in addition to the state-of-the-art privacy-preserving techniques such as homomorphic encryption [20], which may only guarantee protection during training.

## 2. Materials

### 2.1. Existing Research Direction

Architectural patterns for the design of FL were systematically reviewed based on a theory of pattern forms [15]. The patterns include model co-versioning registry, deployment selector, multi-task model trainer, and heterogeneous data handler and aggregator. Moreover, client registry systems based on blockchain technology are proposed for these patterns to maintain participants and their model updates in the FL process. It is identified to be essential to solve non-IID issues, deploy specific models to clusters, address privacy leakages, trace model quality, and detect dishonest client devices [15]. Despite that, blockchain does not align well with the needs of the health care sector and the confidentiality of health data. For instance, it conflicts with protection regulations such as GDPR due to the immutability of stored (metadata) information and could affect P2P PHS features for users to access their PHS offline [1]. Furthermore, BrainTorrent [27] is a serverless P2P FL conceptual framework for highly decentralized environments, specifically for medical centers where medical data are pooled (e.g., hospitals, as in our recent study [28]). Those frameworks are more effective and suitable with a smaller number of participating parties (e.g., where the number of selected participants is not significantly larger than training samples in the participants’ devices), rigid infrastructure, and existing trust relationships.

In order to achieve a satisfying global model (with higher accuracy and convergence speed), FL requires iterative training at the edge and frequent model aggregations in the cloud due to highly imbalanced data and complex model structures. On P2P PHSs, available data points at the edge differ considerably across patients, $\forall e\neq \;\bar{e};\;e,\;\bar{e}\in Clients$. Therefore, prediction loss on $\left(x_{i},y_{i}\right),$ yields $f_{e}\left(\omega \right)\neq \;f_{\;\bar{e}}\left(\omega \right)$. This leads to prohibitive network communication overhead by requiring more training rounds to achieve a model with good accuracy and FL performance [20,23,24]. Model update compression, sampling, and algorithm optimization are used to reduce the aggravating overhead [20]; however, such techniques could cause information loss and performance degradation, among others [20,23,24]. As such, FEDn [23] and SHARE [24] leverage hierarchical design settings to make FL more scalable with enhanced communication efficiency. They randomly select subsets of distributed edge nodes to aggregate their associated neighbors’ models (one node to one aggregator) and commit the models to a cloud aggregator. We argue that the edge aggregators should be deterministically selected to ensure a trusted computing base and required computational device resources. 

The P2P PHS architecture has many tiers; the practitioners (super peers) act as managers of their patients (normal peers) and each patient could be associated with more than one practitioner. Due to the patient–practitioner trust relationship, the super peer nodes would be leveraged for the edge node model aggregation in our FL design. Another FL framework used a hierarchy of information (public and private) and client identity information as part of features to cluster and aggregate private information from inconsistent clients to determine their peculiarity with other clients and train and deliver the personalized model to clients [26]. Nevertheless, such solutions relied on client–server architecture. Further, they did not focus on the inclusion of clients’ identity verification services in the training process for a simple cause: current studies [15,22,23,24,25,26] focus on FL cases, where the datasets at the edge devices overlap in a sample and/or in feature spaces, learnings are to produce a single global model, and trusted communication is assumed to be established for the training.

### 2.2. Federated Learning Technical Definition

There exist various design patterns of FL [15,22,25,29]. A typical FL goal for a non-convex neural network optimization, as demonstrated [30], is to minimize a global loss function f of the weighted average of each participating edge device’s losses (Equation (1)).

(1) $$f\left(\omega \right)=\overset{K}{\underset{e=1}{\sum }}\frac{n_{e}}{n}F_{e\;}\left(\omega ,{\;P}_{e}\right)$$

where K is the number of participating edge devices, e is the index for individual devices, $P_{e}$ is the local dataset on edge device *e*, $n_{e}=\left|P_{e}\right|$ is the number of data points that edge device e contributes to the training, $n=\sum n_{e}$ is the total number of data points, and $F_{e}\left(\omega ,\;P_{e}\right)$ is the loss of the model with weights ω calculated on the data of edge device e.

The typical workflow is as follows: (i) a central server orchestrates the training process and sends a pre-trained model to the sample *m* of fraction *C* of participating edge devices (selected randomly) from *K* devices for further training (Algorithm 1); (ii) each participating edge device *e* locally trains the model (e.g., using SGD) for *E* iterations/epochs using the local data $P_{e}$ and computes the local training loss using $F_{e}\left(\omega ,\;P_{e}\right)$ and then sends the training parameters back to the central server for aggregation; (iii) the central node aggregates all the models received from the edge devices—the weighted sum of model parameters *ω*; (iv) the central node sends the aggregated global model to the edge devices; (v) the devices replace the local model with the new one received and use it for local predictions or further training.
**Algorithm 1** FL Algorithm with a central server—FedAvgCentral ServerEdge devicesω: Global model weights $\omega_{t}$: Global model weights at time *t* K: number of edge devices 0≤C≤1: fraction of participating edge devices *m*: number of participating edge devices $e\in S_{t}$: index of a participating edge device|B|: batch size $P_{e}$: local data on device *e* E: number of training epochs $\nabla F_{e\;}\left(\omega ,\;b\right)$: gradient of the loss with respect to batch *b* α: learning rate
**orchestrate function** () { initialize *w*_o_ **for** (each round *t* = 1, 2, 3…) {   *m* ← max (*C*, *, *K*, 1);   *S_t_* ← (random set of *m* participating edge devices);    **for** (each device *e* ∈ *S_t_* **in parallel**) {     
$\omega_{t+1}^{e}\leftarrow \;$ update function
$e,\;\omega_{t}$;                     }    
$\omega_{t+1}\leftarrow \;\sum_{e=1}^{K}\frac{n_{e}}{n}\omega_{t+1}^{e}$;                }             }**update function** (*e*, *w*) { *β* ← (split *P_e_* into batches of size
|B|
**for** each local epoch *i* from 1 to *E*) {
  **for** (batch *b* ∈ *β*) {      
$\omega \;\leftarrow \;\omega -\alpha \nabla F_{e\;}\left(\omega ,\;b\right)$          }               } **return**
*ω* to central server          }


### 2.3. Envisioned Health Care Systems Paradigm

**Figure 1:**
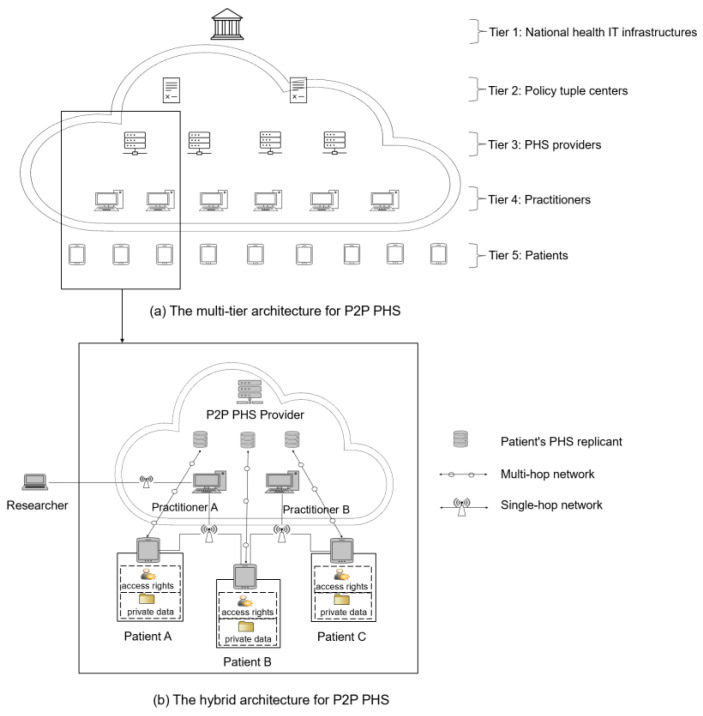
Peer-to-peer (P2P) high-level architecture for patient-centered health care information systems (PHSs) [1].

P2P PHSs (e.g., Doc.AI [12], COVID-19 contact tracing systems such as German Corona Warn App [31]) are promising health care information systems that reap the benefits of decentralization to offer improved privacy management, data sovereignty, and resilience to single points of failure, a new paradigm shift as described by Alex Pentland et al. [6,32]. Our previous studies [1,6] and P2P PHSs such as Doc.AI brands [12] demonstrate that P2P PHSs are feasible. They could be suitably deployed on the multi-tier structure and hybrid P2P PHS architecture (Figure 1), in which the P2P PHS network is an overlay of the model hierarchical relationships between national IT infrastructures such as German HTI and tuple centers—e.g., Health Information Exchanges that make EHRs standardized and interoperable based on HL7 FHIR [33]—tuple centers and PHS providers, PHS providers and practitioners, and practitioners and patients [1]. These actors and their relationships support an ecosystem that provides a collaborative process of trust and interoperability based on existing health care systems and abilities such as registration and identity verification, enforcement of data regulations, scalability, and resource availability.

Tier 1 includes national IT infrastructures (Figure 1) that manage and certify various PHS providers and define and enforce implementations of data regulations and ontologies for sharing health records on PHS networks. In Tier 2, agent-based coordination models are used to facilitate data semantics, user lookup services, and a medium for sharing health records between various PHS providers in a P2P manner. However, a P2P PHS is limited to Tier 2’s PHS subscribers. Tier 3 includes P2P PHSs (e.g., hospitals or insurance companies). They facilitate sub-peers’ inter-communication in the network while providing additional services such as a user-centric backup option or emergency access via a separate private cloud server (Figure 1). Patients could leverage the backup feature to store a replica of their local storage (P2P PHS) and share their stored data with others remotely via multi-hop networks. 

Tier 4 contains the health practitioners, and each PHS provider could have many practitioners in their network. A practitioner associates and manages a group of patients and their public identities for lookup and data access, for instance under the control of distributed hash tables (DHT) [34]. Moreover, patients could be associated with multiple practitioners (e.g., a family doctor and a neurologist) and P2P PHS providers (e.g., PHS for tracking opioid disorders and a PHS for monitoring mental wellness). These PHSs could communicate if they subscribed to a common tuple center (Tier 2, Figure 1). This way, a patient’s family doctor could access the patient’s remote data stored at the neurologist’s PHS cloud server for diagnosis if the patient grants access rights. Note that the nodes in Tiers 1–4 differ from centralized servers used for FL [1]; e.g., these nodes act as federated super peers and are not as powerful as centralized servers. They only act as managers (facilitate resource sharing and downloading, computations, etc.) of patients whose data are stored initially locally in their P2P PHSs.

Tier 5 comprises the patients, and they are in absolute control of their local P2P PHS, and the replica of their data stored at the providers’ cloud servers. Patients can grant access to their data via single-hop (e.g., WIFI direct) or multi-hop wireless networks [1,6]. Parties such as researchers or even PHS providers looking for data for research purposes or providing additional features can request access to patients’ data through the practitioners’ PHS networks. The request is then forwarded to the patients for approval. To ensure privacy, the learning should be carried out (if accepted by patients) by leveraging FL techniques on the local P2P PHS device or a replica of patients’ data stored at the providers’ cloud server—concrete and useful learning scenarios for this study are exemplified below.

### 2.4. Exemplary Learning Case

A PHS provider (or a researcher) that would like to provide or train health care ML models for patients using P2P PHS would need to rely on heterogeneous data distributed across patients’ devices and possibly additional data sources, e.g., from hospitals. In most cases, a patient could not have the required data or enough data at the edge for the model learning, but such a health care model would be significant for the patient’s health. For example, patient Y needs models to monitor his hypertension and potential risks of diabetes, and patient X needs models to track his diabetes and risks of getting hypertension—models are offered by the PHS provider. This way, the data (or rather some features) from one patient could be useful to the other to train global and personalized models, e.g., for predicting diabetes and hypertension risks, albeit the participants’ datasets have small intersections (i.e., they differ in samples and feature space). Patient X’s data features such as age, systolic and diastolic blood pressure (BP), daily activities (e.g., step count), and family disease history could be useful for the training of the model, which patient Y needs, to detect relevant abnormal health conditions.

**Figure 2:**
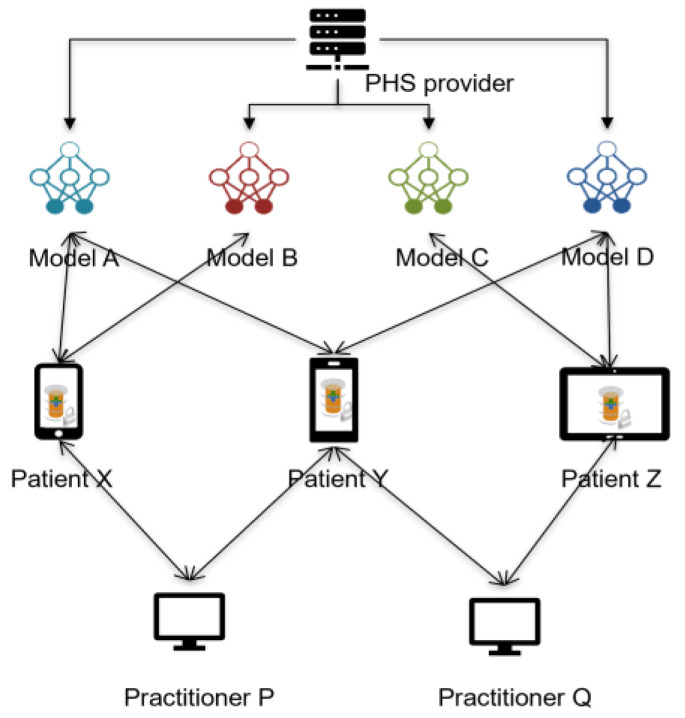
PHS provider offers multiple personalized health care models (tracking of pain, opioid disorder, etc.) to patients on their P2P PHS client software. Every patient could subscribe to any relevant models, and each model depends on several heterogeneous features (or data)—represented with different colors—from other patients and other sources for the training.

The design of P2P PHS necessitates using FL to train these models, as the data are stored locally at the edge and cannot be pooled at a central node for training due to concerns such as privacy [22]. Moreover, the fundamental P2P PHS features also affect the model training; for instance, the learning orchestrator (e.g., a researcher) has neither access nor direct communication with the patients. Therefore, leveraging entrusted roles such as practitioners in the architectural hierarchy (Figure 2), who have mutual relationships with the patients, is essential for requesting model training on the patients’ local storage. Health practitioners’ nodes in the network could act as heterogenous data handlers to improve FL communication efficiency and performance (detailed in Section 3). The FL study could also be conducted at the practitioners’ nodes when the patients grant access rights (for instance, to patients’ P2P PHS replicants at the hospital cloud server—Figure 1).

## 3. Results—Novel Federated Learning Framework

**Figure 3:**
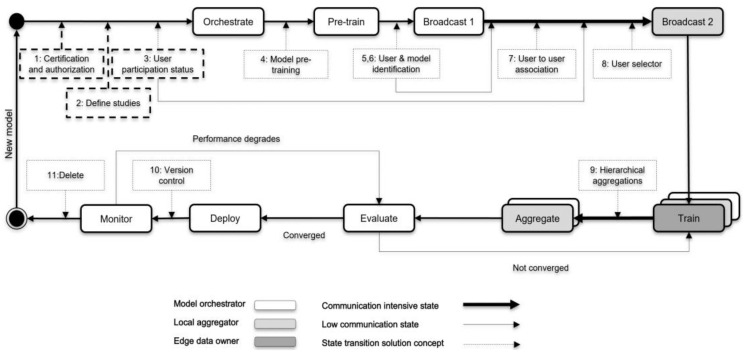
A model life cycle for the FL on hierarchy and hybrid models (patient-centered health care information systems) building on the current trust structures of health care systems—CareNetFL.

Given the architecture (Figure 1) and the learning scenario (Figure 2), we propose the following concepts to achieve a suitable but trustworthy FL on P2P PHS networks (Figure 3), CareNetFL. Alongside the model, the additional concepts function as algorithms (which could exist as a technical module, e.g., certification checker) that would be part of or integrated into the FL process.

### 3.1. Phase 1: Certification and Authorization

Before a new FL model/algorithm (as exemplified in Section 2.4) is orchestrated to the network (e.g., by a scientist or hospital), it should conform to certain requirements (regulations such as GDPR, etc. [17]) and be certified and authorized by trusted bodies (e.g., Technical Inspection Association in Germany, TÜV). Next, the model orchestrator can obtain access credentials such as a token and digital certificate to make the first registration on the PHS network. This registration allows the model orchestrator to receive an identity for their model and other technical specifications, such as API access credentials (URL, smartcard, PIN, etc.). Once such conditions are met, the model could be initiated to the P2P PHS network for training guided by the following sequential phases (see Figure 3).

### 3.2. Phase 2: Define Studies

The study to be orchestrated should be deemed suitable to be run on the network. Initially, a PHS provider could define the list of allowable studies that could be run on the network among the list of models that are authorized in phase 1. For instance, a model for predicting the risk of diabetes could be only registered for training if patients suffer from the such disease, i.e., an ophthalmic model to an ophthalmic clinic and a neurological model to a neurological clinic. PHS providers such as hospitals could perform further checks on the ML model to ensure it meets specific requirements or basic outline perspectives (compliance with security and data protection laws) before it is allowed in the network.

### 3.3. Phase 3: User Participation Status

Once the PHS provider approves the study, the learning orchestrator starts searching for participants (practitioners and patients) who want to partake. In addition, these concepts necessitate keeping track of participants’ public identities (un-linkable to users’ original identities to avoid privacy leakage) and their consent to participate in the FL training of any defined study. The model orchestrator could derive knowledge of whether the volume of data available from the size of interested participants would be sufficient for the model training.

### 3.4. Phase 4: Model Pre-Training

Models can optionally be pre-trained if similar non-private data are available from secondary data sources. Model pre-training can speed up federated learning, enable models to achieve a better performance, and reduce the impact of data heterogeneity on federated learning [35]. Before initiating the model training in the network, the model could be pre-trained using secondary data sources (this could also be provided by the PHS provider) to reduce the need for large datasets to achieve a personalized or generalized model with higher accuracy.

### 3.5. Phase 5 and 6: User and Model Identification

In this phase, users (patients and practitioners) who declared their interest in participating in the FL process are pre-selected using their public/pseudo identifiers, which cannot be reverse-engineered to their actual identities. This public information can be managed and accessed through, for instance, the PHS provider’s network (Figure 1). The model (or study) identity would be linked/mapped with the learning participants to deliver appropriate models to patients, offering traceability and purging contributions from malicious entities in the network and maintaining model versions. For instance, Doc.AI [12] leverages the client registry server to ensure updates are applied to the right version of the model [15]. Additionally, user-to-model mapping enables early stopping in situations where a local model overfits [15]. Keeping records of patients’ subscriptions is essential when there is a higher number of patients subscribing to many models (e.g., as provided by the PHS provider—Figure 2) that also have correlated data features on their P2P PHSs. In addition, patients’ identities need to be maintained to match their actual devices and pseudonyms and their practitioners for model update aggregation, matching, and deploying individualized models. Hence, it is essential to leverage the user authentication system and incorporate it into the FL life cycle training process.

Once the identification phase is achieved, a broadcast message is sent to local aggregators’ nodes (practitioners) in the network. Broadcast 1 is only to instances that might have functional IT infrastructure (e.g., ten practitioner nodes), and hence adds little overhead to the network.

### 3.6. Phase 7: User-to-User Association

Since practitioners could have access to patients’ partial data, clustering patients based on their practitioners is essential before selecting participating and eligible patients to understand if a single patient is suitable for the study. Establishing this linking ability allows practitioner nodes to pre-select eligible patients with the data needed for the training. Grouping patients based on disease relevance could reduce the heterogeneity of the data. A patient could be under practitioner A due to her disease X and practitioner B due to her disease Y (see Figure 2). This means that a patient could be associated with multiple practitioners; however, the association only creates a link for the specific data type that a practitioner deals with and is permitted to be accessed by the patients. Suppose more than one practitioner has equal access to a patient’s health data. In that case, the concept only assigns the association link (randomly or defined beforehand by the patients) to one of the practitioners to act as a relay. This association mitigates the likelihood of aggregating model parameters (weights) emerging from the same data sample of a patient. In contrast, this concept exposes (meta) information to unselected practitioners (nodes) about their patients in possession of specific data needed for the training. Nonetheless, the patients chose a different practitioner node in the network to associate their data for this FL process. In this context, users should be transparently informed about what data and meta information are shared and that they can opt out of the model training if they do not want to leak such information.

### 3.7. Phase 8: User Selector

Patients’ (meta) data conformance to the model training requirements (e.g., age, gender) could be determined at this stage. This selection process could be performed with the meta information (as authorized/consented by patients) available at the practitioners’ tier or on P2P PHS replicants (Figure 1). A patient with no health data needed for the model training will not be selected for the training but could receive the final trained model for usage at the edge (when s/he subscribed to that). Next, practitioner nodes forward the pre-trained model to the selected edge/local devices (P2P PHSs) of patients for training. Note that applications of the proposed framework need to consider selection bias issues that may occur during user selection.

### 3.8. Phase 9: Hierarchical Aggregation

**Figure 4:**
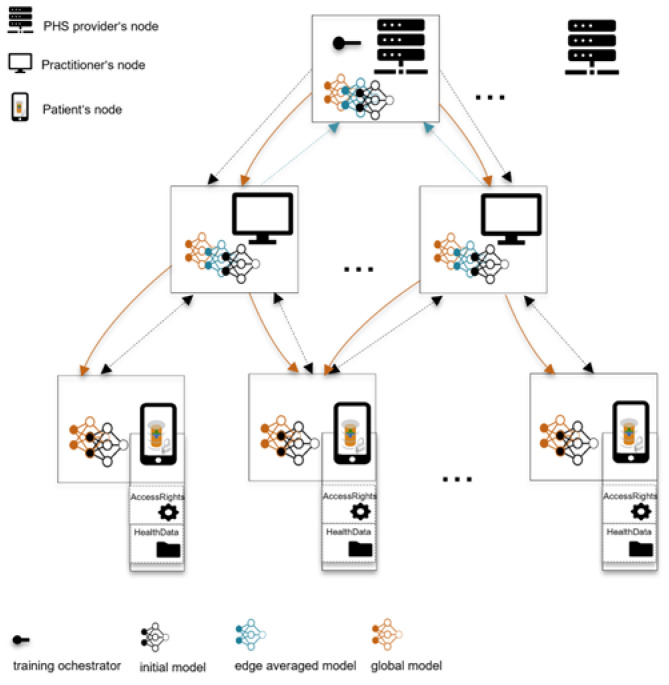
ML model training and hierarchical aggregation of model parameters. (i) PHS provider (global aggregator) orchestrates the training process and sends an initial model to practitioner nodes (local aggregators), (ii) local aggregators send the initial models to their patients’ edge nodes, (iii) edge nodes perform a few training epochs and commit model updates to the practitioners’ nodes, (iv) local aggregators aggregate local models of their clusters and commit them to the global aggregator, (v) global aggregators aggregate edge models and forward the global model to local aggregators, (vi) local aggregators send the global model to patients’ edge nodes for prediction or further training.

At the edge nodes, the model is trained for a few epochs with increased user privacy—e.g., using techniques such as differential privacy (DP) and secure multi-party computation (SMPC)—as shown in Figure 4. The global and local model aggregations could be synchronous by waiting for model updates from enough participants or asynchronous by skipping delayed model updates. The former reduces model biases and increases personalization, while the latter improves aggregation latency. The model is evaluated, for instance, at the model orchestrator instance/node, until a higher-performing model is achieved, which also meets the defined model utility requirement—performance, speed, accuracy, etc. A model orchestrator could drop participants during training based on pre-defined criteria (e.g., data quality, computational power, or model performance at the edge [30]) to reduce communication latency and increase security and model quality. However, such criteria could affect the model’s personalization/generalization and performance and yield privacy issues due to access to users’ device computational resources.

Note that we do not focus on any specific aggregation methods and preserving–enhancing techniques. Instead, we envision future users of a concrete implementation of our framework to experiment with different state-of-the-art algorithms for aggregation and preserving user privacy.

The final model could then be deployed to the patient devices and/or to practitioner devices for their usage (e.g., prediction for risk of hypertension). At this stage, matching occurs, ensuring patients only obtain the final subscribed FL model(s). If this is a study, e.g., by a researcher who conducts the study with no deployment intention or plan, the matching process is skipped. Due to regulatory requirements (e.g., GDPR), the model orchestrator could share the result with the PHS providers or rather with patients directly in a suitable form. Additionally, the initial intention of FL model training needs to be defined in phases 1 and 2. For instance, researchers’ goals might be to train the model but not share the results or deploy them for patients’ usage.

### 3.9. Phase 10: Version Control

At the time of model deployment, the model is stored and managed with the metadata (user-to-user association, etc.) acquired via the previous concepts or phases (Figure 3). The framework allows for constant monitoring to ensure privacy, trust, security, and performance. If a model performance degrades, the training phases are re-executed until a new converged and highly performing model is obtained. The old and new model versions are maintained at the provider’s network for traceability and ease of re-deployment to the edge devices. A patient unable to download the updated model early due to, for instance, a lack of bandwidth or space may have the chance to download the newer version next time in the P2P PHS client software—in the case of a trained model for users’ usage.

### 3.10. Phase 11: Delete

An ML model being trained for research purposes could be deleted when the training circle is completed, and the model achieved a satisfying performance—it might be necessary for the study results to be shared with the participants due to regulatory requirements. A model orchestrated and trained to be deployed and used on the P2P PHS network can also be deleted when its performance degrades or when issues such as data privacy leakage or security vulnerabilities are detected. Nonetheless, the learning orchestrator could train a new model (e.g., a new release or version of the deleted model) following the described phases one to ten (Figure 3).

## 4. Discussion

The health care ecosystem is not a zero-trust environment. For instance, according to a survey from the University of Pennsylvania in 2020, more than half of 3543 participants were less willing to share their EHR for health-related uses or research purposes due to underlying trust and privacy concerns [36]. However, it is sufficient for trusted parties such as practitioners involved in patient care to have a consistent view of patients’ health data/status, which patients already share in the current health care process. Therefore, instead of selecting any anonymous/random nodes in the network for model update aggregation (comparable to the current study [24]) that could pose privacy or regulatory concerns [37], such entrusted relationships between parties are leveraged in our FL design for P2P PHS.

In this paper, we propose CareNetFL, an FL conceptual framework (Figure 3) that builds on the existing health care trust structure and is suitable for the emerging P2P PHS multi-tier structure and hybrid architecture (Figure 1) with increased trust, scalability, and security. We define specific roles such as PHS providers that provide ML-based e-health services as learning orchestrators (and global aggregators), practitioners as local model aggregators, and patients as edge data owners in order to align stakeholders’ objectives and integrate them into the FL process. Since data are stored locally at the edge under the sovereignty of patients, the super nodes (local and global aggregators) only act as managers of their patients and are not as powerful as a central server [1]. Our FL concepts ensure that data owners’ nodes are associated with their local aggregators’ nodes, that local aggregators’ nodes are associated with a model orchestrator, etc. This way, data owners commit their local model updates to their entrusted local aggregators’ nodes—practitioners involved in their care. Local aggregators perform a few training rounds and then commit their combined local models to the global aggregator for global aggregation. Local aggregations at the edge via practitioners ease patient data access and reduce the frequency of committing local models directly to the global aggregator [38]. The inclusion of medical practitioners in the FL process serves as an enabler for the practitioners to align their needs in the application life cycle. It allows for practitioners to oversee the diagnostic criteria of the model and to address the barriers (unfamiliar data structure, missing measurements, etc.) to using patient-generated data across clinical settings [20,39]. 

CareNetFL is suitable and advantageous for P2P PHS since client models’ updates are aggregated in a hierarchical decentralized approach. Contrasted with the typical FL with a central server (e.g., a recent study [37] shows a curious central server that actively fakes model updates of participating clients to reconstruct the individual training data of a client, even when using secure aggregation), in our FL setting, it is certain that the local aggregators (practitioners) can be trusted and will not actively become malicious. Challenges of FL with a central server are known [20,37]. However, the absence of a single entity in the network in CareNet would not cause the single-point failure nor affect the training process.

Further, the hierarchical averaging of model updates improves parallelism and scalability since local aggregators aggregate local models independently. In addition, grouping patients based on their practitioners (e.g., dementia patients to neurologists) means bringing data close to a uniform distribution. Performing a few training iterations at the edge reduces the required communication rounds to train a model with higher accuracy. Previous hierarchical FL studies [23,24] demonstrate clustering data based on relevance to reducing the training rounds needed to achieve a higher-performing model. However, such studies are not based on highly heterogenous data such as medical data. Furthermore, they used random entities for model update aggregation, which could pose privacy concerns. They used blockchain for client management, which is unfit for health information systems [1]. In addition, we chose an appropriate learning case where heterogeneous data across edge devices are required for the FL, and individual participants could subscribe to any relevant offered e-services in their P2P PHS. Aptly, PHS providers (e.g., hospitals) could use our CareNetFL to provide an FL framework on their network while maintaining user privacy, usability, and performance.

In our conceptual FL (Figure 3), nodes on P2P PHS need to be identified to be associated (e.g., which patients are under which practitioners). Consequently, our concept focuses on leveraging an identity-based authentication system to verify model updates end to end and associate nodes with others (patients with their practitioners) accordingly on the P2P PHS network for the FL process. Our solution could provide a secure user reidentification feature for delivering individualized models to the edge. It eases the ability to deploy to the existing authentication services of P2P PHS through session variables’ validation and migration to the security measures to be provided by extensive IT infrastructure such as the German HTI. Authentication mechanisms would also be used for the node-to-node association on P2P PHS network for the design of FL algorithms but also to enhance security and accountability. Existing FL frameworks [15,20,22,27,40] assume trusted communication is in place before and during the training. For example, a study by Bonawitz et al. [22] excluded the need for identity-based authentication in FL because participants were required to be anonymous. Despite that, they still relied on a third-party service to ensure that only genuine devices participated in the FL process. Data anonymization reduces system accountability and is undesirable for P2P PHSs since patients’ identification (non-reverse-engineerable to patients’ original identities) could be leveraged to deliver personalized health care—clients need to be identified to match and deploy specific subscribed models for personalized prediction or inference [15]. 

FL requires authentication, transport encryption, and other verification mechanisms to secure this aspect of the underlying infrastructure. Failure to implement these can make it easy to launch attacks on FL systems (e.g., model poisoning [15,20]) and effectively deter the FL’s goal and patients’ well-being in the case of P2P PHSs due to the sensitivity of medical data. DP, SMPC, and HE ensure that the model and data remain secure [20] but only during training. Although such techniques could accept any input (malicious or not), they cannot verify the source from the participating devices/users. Consequently, malicious clients could easily join the FL training process and disrupt the training by providing corrupted updates to the aggregation server. With secure authentication mechanisms integrated into FL, the chance of including malicious users is reduced, providing legal accountability in case malicious actions are detected, mitigating the vulnerability to data and model poisoning attacks, and improving the effectiveness of the privacy-preserving techniques employed.

This study focuses on a conceptual design for an FL system specifically for user-centric decentralized information systems deployed at the edge, P2P PHSs. Accordingly, how the choice of ML training technical requirements will affect FL performance is beyond the scope of this study. This study’s further direction will consider implementing the core concepts of the proposed FL framework and conducting suitable experiments using suitable datasets and security, privacy, and evaluation mechanisms. The following drawbacks are part of the considerations in future research: concepts such as identity authentication mechanism, user-to-user association, user-to-model mapping, model versioning, and edge devices’ model update management are implemented at the PHS provider network. They will still demand substantial computational power and other resources to be well integrated with the FL framework. Although practitioners are leveraged in the FL process to ease access to patients’ data and align their needs, privacy leakage could occur when the learning orchestrator access patients’ metadata and training model parameters for selecting appropriate users to participate in the FL.

## 5. Conclusions

Aligning health care stakeholders’ needs in the FL process for health care information systems could make it more collaborative, increase trust, and mitigate barriers to adopting AI solutions and patient-generated data in medical workflows. In this manuscript, we share our perspective on how such a need (that exists in the current health systems) can be integrated with the FL process for the emerging decentralized health care information systems deployed at the edge. Our novel conceptual FL framework, CareNetFL, allows the data to remain at the edge (where they are located) and to maintain privacy and fundamental features of P2P PHSs. It further leverages entrusted parties (practitioners’ nodes in the network) to serve as a model update aggregator and a medium for requesting model training on patients’ local storage. This way, practitioners engage in the learning process and alleviate security and privacy concerns compared to systems that leverage any random entities for model update aggregation in the FL process [37]. Additionally, the proposed end-to-end FL framework could function as a driver to reduce the imbalance and non-IID data distributions and system heterogeneity challenges of FL at the edge, mitigating communication latency and improving FL performance. Our manuscript shares that FL can potentially address hurdles concerning privacy and the egress of sensitive medical data away from data owners [29]. However, FL is not a one-size-fits-all solution. The system architecture is key to FL design, deployment, and performance in real-world environments. The envisioned future direction for this study aims to implement CareNetFL with its core features and suitable data samples, and then compare a trained model’s performance with CareNetFL and appropriate benchmarks. 

## Data Availability

No new data were created or analyzed in this study. Data sharing is not applicable to this article.
